# 1700. Efficacy and Safety in Subjects with Renal Impairment in Contezolid and Contezolid Acefosamil Phase 2 and Phase 3 Skin Infection Clinical Trials

**DOI:** 10.1093/ofid/ofac492.1330

**Published:** 2022-12-15

**Authors:** Edward Fang, Huahui Yang, Hong Yuan

**Affiliations:** MicuRx Pharmaceuticals Inc, San Carlos, California; MicuRx Pharmaceuticals Inc, San Carlos, California; MicuRx Pharmaceuticals Inc, Foster City, CA, USA, shanghai, Shanghai, China

## Abstract

**Background:**

Contezolid (CZD; MRX-I) is a novel oral (PO) oxazolidinone with potent activity against Gram-positive pathogens, including methicillin-resistant *Staphylococcus aureus* (MRSA) and vancomycin-resistant *Enteroccocus* (VRE). Contezolid acefosamil (CZA; MRX-4) is an intravenous (IV) double prodrug of CZD. Nonclinical and initial clinical data indicate CZA and CZD may cause less myelosuppression, particularly with longer duration therapy, and with reduced risk of monoamine oxidase inhibition compared with linezolid (LZD). In 3 CZD Phase 2 (Ph2) and Phase 3 (Ph3) skin infection trials and 1 CZA Ph2 acute bacterial skin and skin structure infection (ABSSSI) study, primary efficacy and overall safety outcomes were comparable to LZD, and the most common treatment emergent adverse events (TEAEs) were gastrointestinal; however, hematologic laboratory abnormalities and TEAEs were less common with CZD and CZA. In June 2021, CZD was approved in China for complicated skin and soft tissue infections (cSSTI). Sequential therapy with CZA IV followed by CZD PO is being evaluated in global Ph3 diabetic foot infection (DFI) and ABSSSI clinical trials. Because patients with diabetes commonly have diminished kidney function, efficacy and safety outcomes in subjects with renal impairment were evaluated in completed Ph2 and Ph3 CZD and CZA studies.

**Methods:**

In 4 CZD and CZA Ph2 and Ph3 skin infection trials, subjects were included with estimated creatinine clearance (CLcr) of 60 to < 90 mL/min (mild impairment) and 30 to < 60 mL/min (moderate impairment); no dose adjustments were made for renal function status. Primary efficacy outcomes and occurrence of TEAEs were evaluated for CZD and CZA subjects with no (CLcr ≥90 mL/min), mild, and moderate renal impairment.

**Figure:**
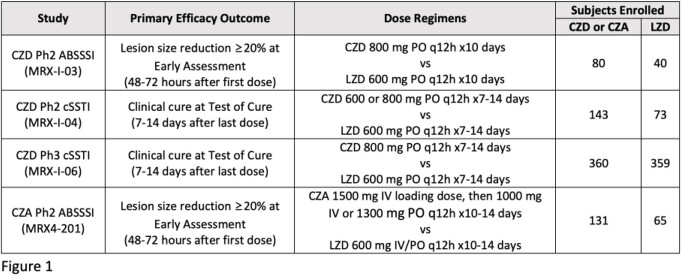

**Results:**

Primary efficacy outcomes and occurrence of TEAEs for CZD and CZA subjects with mild or moderate renal impairment were similar to that of subjects with no impairment in 4 skin infection trials.

**Figure:**
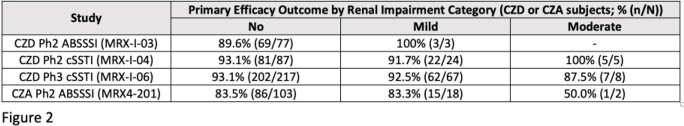

**Figure:**
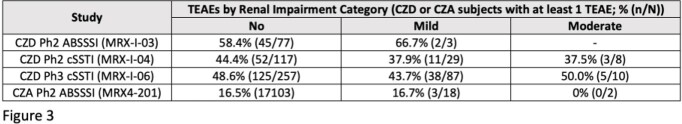

**Conclusion:**

In 4 completed Ph2 and Ph3 skin infection clinical trials, subjects who received CZD or CZA with mild and moderate renal impairment appeared to have primary efficacy and safety outcomes similar to subjects with no impairment, supporting current Ph3 global DFI and ABSSSI studies which will enroll subjects with reduced kidney function.

**Disclosures:**

**Edward Fang, MD**, MicuRx Pharmaceuticals Inc: Employee **Huahui Yang, MS**, MicuRx Pharmaceuticals Inc: Employee.

